# Supplementary material for: The transcriptional and phenotypic characteristics that define alveolar macrophage subsets in acute hypoxemic respiratory failure
Source: Nat Commun. 2023 Nov 17;14:7443. doi: 10.1038/s41467-023-43223-0 (PMC10656558; doi:10.1038/s41467-023-43223-0)
Supplement: Supplementary file 1 — Supplementary Information [file 41467_2023_43223_MOESM1_ESM.docx]

**Supplementary Information**

**The Transcriptional and Phenotypic Characteristics that**

**Define Alveolar Macrophage Subsets in Acute Hypoxemic Respiratory Failure**

**Authors**: Eric D. Morrell,^1†^* Sarah E. Holton,^1†^ Matthew Lawrance,^2^ Marika Orlov,^3^ Zoie Franklin,^2^ Mallorie A. Mitchem,^2^ Hannah DeBerg,^2^ Vivian H. Gersuk,^2^ Ashley Garay,^1^ Elizabeth Barnes,^1^ Ted Liu,^1^ Ithan D. Peltan,^4^ Angela Rogers,^5^ Steven Ziegler,^2^ Mark M. Wurfel,^1^ Carmen Mikacenic^2^*

**Affiliations**:

1. Division of Pulmonary, Critical Care, and Sleep Medicine, University of Washington, Seattle, WA, USA

2. Translational Immunology, Benaroya Research Institute, Seattle, WA, USA

3. Division of Pulmonary Sciences and Critical Care Medicine, University of Colorado, Aurora, CO, USA

4. Division of Pulmonary and Critical Care Medicine, Intermountain Health, Murray, UT, USA

5. Division of Pulmonary and Critical Care, Stanford University, Stanford, CA, USA

† These authors contributed equally to this work

* Corresponding Authors

**List of Supplementary Materials**

**Table S1** – Study Inclusion and Exclusion Criteria

**Table S2** – Description of Transfused Blood Products

**Table S3** – CITE-seq Antibody Panel

**Table S4** – Quality Control Metrics of CITE-seq Data

**Table S5** – Top 20 Marker Genes for Each Alveolar Myeloid Cluster

**Table S6** – Summary of Published Datasets Compared with Current Dataset

**Table S7** – Characteristics of Participants with or without ARDS

**Table S8** – Top 20 Marker Genes for “Integrated” Blood-BAL UMAP

**Table S9** – Most Discriminatory Cell-Surface Proteins Between Alveolar Monocyte and Macrophage Clusters

**Table S10** – Flow Cytometry Cell-Surface Antibody Panel

**Figure S1** – Clinical and Biomarker Trajectories of Enrolled Participants

**Figure S2** – Distribution of the Number of Genes per Cell Detected for Each Participant

**Figure S3** – Distribution of the Percentage of Reads Mapping to Mitochondrial Genes per Cell for Each Participant

**Figure S4** – Associations Between Alveolar Myeloid Subsets and Biomarker Levels

**Figure S5** – Associations Between Alveolar Myeloid Subsets and Clinical Severity

**Figure S6** – Associations Between Alveolar Myeloid Subsets and Age

**Figure S7** – RNA Velocity of Alveolar Monocyte and Macrophage Subsets

**Figure S8** – Integrated Blood-Lung Myeloid Cell Clustering

**Figure S9** – Cell-Surface Antigen Specificity Scores

**Table S1. Study Inclusion and Exclusion Criteria**

| ***Inclusion Criteria*** |
| --- |
| Mechanically ventilated through endotracheal tube |
| Risk factor for ARDS: pneumonia, sepsis, trauma, aspiration, or massive transfusion |
| Intubated < 7 days |
| Radiographic infiltrate |
| F_i_O_2_ ≥ 25% |
| Legal next of kin able to provide informed consent |
| ***Exclusion Criteria*** |
| < 18 or > 85 years of age |
| Pregnant |
| Prisoner |
| Positive SARS-CoV-2 PCR test |
| Charlson comorbidity pulmonary risk index score > 0 (dyspnea at rest or with mild exertion, chronic supplemental oxygen use, or chronic hypercapnia) |
| Known history of COPD, emphysema, or chronic bronchitis |
| Known history of interstitial lung disease |
| Known history of asthma |
| Known use of home respiratory support (except use for sleep apnea) |
| Known severe pulmonary hypertension (mean PA pressure > 55 mm Hg) |
| Mean arterial pressure < 55 mm Hg while supported on vasopressors |
| Intracranial pressure > 20 mm Hg |
| Unstable angina or acute MI |
| AIDS with CD4 < 200 |
| Bone marrow or solid organ transplant |
| Known metastatic cancer |
| Severe burns (>10% TBSA) |
| Admission for intracranial hemorrhage |
| Legal next of kin not fluent in English or Spanish |

AIDS = acquired immunodeficiency syndrome; COPD = chronic obstructive pulmonary disease; MI = myocardial infarction; PA = pulmonary arterial; PCR = polymerase chain reaction; TBSA = total body surface area

**Table S2. Description of Transfused Blood Products**

| ***ID*** | ***Primary Risk Factor*** | ***Secondary Risk Factors*** | ***# Units PRBCs Transfused*** | ***# Units WB Transfused*** | ***Estimated Total Donor WBCs Transfused*** | ***Recipient WBC (cells/μL)*** | ***Estimated Total Recipient WBC*** | ***% Donor WBCs in Recipient*** |
| --- | --- | --- | --- | --- | --- | --- | --- | --- |
| 1 | Trauma | Contusions/Massive Transfusion | 3 | 3 | 4.7 cells | 9.80 | 4.9x10^7^ | 9.6x10^-8^% |
| 2 | Trauma | Contusions | 5 | 5 | 7.8 cells | 14.16 | 7.1x10^7^ | 1.1x10^-7^% |
| 3 | Trauma | Contusions | 1 | 0 | 0.8 cell | 7.00 | 3.5 x10^7^ | 2.3x10^-8^% |
| 4 | Trauma | Pneumonia/  Contusions | 0 | 0 | NA | 16.34 | 8.2 x10^7^ | NA |
| 5 | Aspiration Pneumonitis | VT/VF Arrest | 0 | 0 | NA | 7.60 | 3.8 x10^7^ | NA |
| 6 | Trauma | Pneumonia/Massive Transfusion | 13 | 4 | 13.2 cells | 13.01 | 6.5 x10^7^ | 2.0x10^-7^% |
| 7 | NSTI/Sepsis | Volume Overload | 0 | 0 | NA | 14.12 | 7.1 x10^7^ | NA |
| 8 | Trauma | Pneumonia | 0 | 0 | NA | 10.89 | 5.4 x10^7^ | NA |

PRBC = packed red blood cell; WB = whole blood; WBC = white blood cell

All blood products at Harborview Medical Center are leukoreduced with a Pall RC-2/RC-3 leukocyte reduction filter. This filter is certified to result in a 4-log reduction of leukocytes in each unit of blood (e.g. a donor unit with 10,000 cells/μL would contain 1cell/μL after leukoreduction).

**Table S3. CITE-Seq Antibody Panel**

| ***Gene Name*** | ***Description*** | ***Clone*** | ***Barcode*** | ***Dilution*** | ***Source*** | ***Cat. No*** | ***Lot No.*** |
| --- | --- | --- | --- | --- | --- | --- | --- |
| CD86 | anti-human CD86 | IT2.2 | GTCTTTGTCAGTGCA | 210 µl | Biolegend | 399905 | B342300 |
| CD274 | anti-human CD274 (B7-H1, PD-L1) | 29E.2A3 | GTTGTCCGACAATAC | 210 µl | Biolegend | 399905 | B342300 |
| TNFRSF14 | anti-human CD270 (HVEM, TR2) | 122 | TGATAGAAACAGACC | 210 µl | Biolegend | 399905 | B342300 |
| PVR | anti-human CD155 (PVR) | SKII.4 | ATCACATCGTTGCCA | 210 µl | Biolegend | 399905 | B342300 |
| NECTIN2 | anti-human CD112 (Nectin-2) | TX31 | AACCTTCCGTCTAAG | 210 µl | Biolegend | 399905 | B342300 |
| CD47 | anti-human CD47 | CC2C6 | GCATTCTGTCACCTA | 210 µl | Biolegend | 399905 | B342300 |
| CD48 | anti-human CD48 | BJ40 | CTACGACGTAGAAGA | 210 µl | Biolegend | 399905 | B342300 |
| CD40 | anti-human CD40 | 5C3 | CTCAGATGGAGTATG | 210 µl | Biolegend | 399905 | B342300 |
| CD40LG | anti-human CD154 | 24-31 | GCTAGATAGATGCAA | 210 µl | Biolegend | 399905 | B342300 |
| CD52 | anti-human CD52 | HI186 | CTTTGTACGAGCAAA | 210 µl | Biolegend | 399905 | B342300 |
| CD3D | anti-human CD3 | UCHT1 | CTCATTGTAACTCCT | 210 µl | Biolegend | 399905 | B342300 |
| CD8A | anti-human CD8 | SK1 | GCGCAACTTGATGAT | 210 µl | Biolegend | 399905 | B342300 |
| NCAM1 | anti-human CD56 | 5.1H11 | TCCTTTCCTGATAGG | 210 µl | Biolegend | 399905 | B342300 |
| CD19 | anti-human CD19 | HIB19 | CTGGGCAATTACTCG | 210 µl | Biolegend | 399905 | B342300 |
| CD33 | anti-human CD33 | P67.6 | TAACTCAGGGCCTAT | 210 µl | Biolegend | 399905 | B342300 |
| ITGAX | anti-human CD11c | S-HCL-3 | TACGCCTATAACTTG | 210 µl | Biolegend | 399905 | B342300 |
| HLA-A | anti-human HLA-A,B,C | W6/32 | TATGCGAGGCTTATC | 210 µl | Biolegend | 399905 | B342300 |
| PTPRC | anti-human CD45RA | HI100 | TCAATCCTTCCGCTT | 210 µl | Biolegend | 399905 | B342300 |
| IL3RA | anti-human CD123 | 6H6 | CTTCACTCTGTCAGG | 210 µl | Biolegend | 399905 | B342300 |
| CD7 | anti-human CD7 | CD7-6B7 | TGGATTCCCGGACTT | 210 µl | Biolegend | 399905 | B342300 |
| ENG | anti-human CD105 | 43A3 | ATCGTCGAGAGCTAG | 210 µl | Biolegend | 399905 | B342300 |
| ITGA6 | anti-human/mouse CD49f | GoH3 | TTCCGAGGATGATCT | 210 µl | Biolegend | 399905 | B342300 |
| CCR4 | anti-human CD194 (CCR4) | L291H4 | AGCTTACCTGCACGA | 210 µl | Biolegend | 399905 | B342300 |
| CD4 | anti-human CD4 | RPA-T4 | TGTTCCCGCTCAACT | 210 µl | Biolegend | 399905 | B342300 |
| CD44 | anti-mouse/human CD44 | IM7 | TGGCTTCAGGTCCTA | 210 µl | Biolegend | 399905 | B342300 |
| CD14 | anti-human CD14 | M5E2 | TCTCAGACCTCCGTA | 210 µl | Biolegend | 399905 | B342300 |
| FCGR3A | anti-human CD16 | 3G8 | AAGTTCACTCTTTGC | 210 µl | Biolegend | 399905 | B342300 |
| IL2RA | anti-human CD25 | BC96 | TTTGTCCTGTACGCC | 210 µl | Biolegend | 399905 | B342300 |
| PTPRC | anti-human CD45RO | UCHL1 | CTCCGAATCATGTTG | 210 µl | Biolegend | 399905 | B342300 |
| PDCD1 | anti-human CD279 (PD-1) | EH12.2H7 | ACAGCGCCGTATTTA | 210 µl | Biolegend | 399905 | B342300 |
| TIGIT | anti-human TIGIT (VSTM3) | A15153G | TTGCTTACCGCCAGA | 210 µl | Biolegend | 399905 | B342300 |
| ***Gene Name*** | ***Description*** | ***Clone*** | ***Barcode*** | ***Dilution*** | ***Source*** | ***Cat. No*** | ***Lot No.*** |
| isotype | Mouse IgG1, κ isotype Ctrl | MOPC-21 | GCCGGACGACATTAA | 210 µl | Biolegend | 399905 | B342300 |
| isotype | Mouse IgG2a, κ isotype Ctrl | MOPC-173 | CTCCTACCTAAACTG | 210 µl | Biolegend | 399905 | B342300 |
| isotype | Mouse IgG2b, κ isotype Ctrl | MPC-11 | ATATGTATCACGCGA | 210 µl | Biolegend | 399905 | B342300 |
| isotype | Rat IgG2b, κ Isotype Ctrl | RTK4530 | GATTCTTGACGACCT | 210 µl | Biolegend | 399905 | B342300 |
| MS4A1 | anti-human CD20 | 2H7 | TTCTGGGTCCCTAGA | 210 µl | Biolegend | 399905 | B342300 |
| NCR1 | anti-human CD335 (NKp46) | 9E2 | ACAATTTGAACAGCG | 210 µl | Biolegend | 399905 | B342300 |
| PECAM1 | anti-human CD31 | WM59 | ACCTTTATGCCACGG | 210 µl | Biolegend | 399905 | B342300 |
| MCAM | anti-human CD146 | P1H12 | CCTTGGATAACATCA | 210 µl | Biolegend | 399905 | B342300 |
| IGHM | anti-human IgM | MHM-88 | TAGCGAGCCCGTATA | 210 µl | Biolegend | 399905 | B342300 |
| CD5 | anti-human CD5 | UCHT2 | CATTAACGGGATGCC | 210 µl | Biolegend | 399905 | B342300 |
| CXCR3 | anti-human CD183 (CXCR3) | G025H7 | GCGATGGTAGATTAT | 210 µl | Biolegend | 399905 | B342300 |
| CCR5 | anti-human CD195 (CCR5) | J418F1 | CCAAAGTAAGAGCCA | 210 µl | Biolegend | 399905 | B342300 |
| FCGR2A | anti-human CD32 | FUN-2 | GCTTCCGAATTACCG | 210 µl | Biolegend | 399905 | B342300 |
| CCR6 | anti-human CD196 (CCR6) | G034E3 | GATCCCTTTGTCACT | 210 µl | Biolegend | 399905 | B342300 |
| CXCR5 | anti-human CD185 (CXCR5) | J252D4 | AATTCAACCGTCGCC | 210 µl | Biolegend | 399905 | B342300 |
| ITGAE | anti-human CD103 (Integrin αE) | Ber-ACT8 | GACCTCATTGTGAAT | 210 µl | Biolegend | 399905 | B342300 |
| CD69 | anti-human CD69 | FN50 | GTCTCTTGGCTTAAA | 210 µl | Biolegend | 399905 | B342300 |
| SELL | anti-human CD62L | DREG-56 | GTCCCTGCAACTTGA | 210 µl | Biolegend | 399905 | B342300 |
| KLRB1 | anti-human CD161 | HP-3G10 | GTACGCAGTCCTTCT | 210 µl | Biolegend | 399905 | B342300 |
| CTLA4 | anti-human CD152 (CTLA-4) | BNI3 | ATGGTTCACGTAATC | 210 µl | Biolegend | 399905 | B342300 |
| LAG3 | anti-human CD223 (LAG-3) | 11C3C65 | CATTTGTCTGCCGGT | 210 µl | Biolegend | 399905 | B342300 |
| KLRG1 | anti-human KLRG1 (MAFA) | SA231A2 | CTTATTTCCTGCCCT | 210 µl | Biolegend | 399905 | B342300 |
| CD27 | anti-human CD27 | O323 | GCACTCCTGCATGTA | 210 µl | Biolegend | 399905 | B342300 |
| LAMP1 | anti-human CD107a (LAMP-1) | H4A3 | CAGCCCACTGCAATA | 210 µl | Biolegend | 399905 | B342300 |
| FAS | anti-human CD95 (Fas) | DX2 | CCAGCTCATTAGAGC | 210 µl | Biolegend | 399905 | B342300 |
| TNFRSF4 | anti-human CD134 (OX40) | Ber-ACT35 (ACT35) | AACCCACCGTTGTTA | 210 µl | Biolegend | 399905 | B342300 |
| HLA-DRA | anti-human HLA-DR | L243 | AATAGCGAGCAAGTA | 210 µl | Biolegend | 399905 | B342300 |
| CD1C | anti-human CD1c | L161 | GAGCTACTTCACTCG | 210 µl | Biolegend | 399905 | B342300 |
| ITGAM | anti-human CD11b | ICRF44 | GACAAGTGATCTGCA | 210 µl | Biolegend | 399905 | B342300 |
| FCGR1A | anti-human CD64 | 10.1 | AAGTATGCCCTACGA | 210 µl | Biolegend | 399905 | B342300 |
| THBD | anti-human CD141 (Thrombomodulin) | M80 | GGATAACCGCGCTTT | 210 µl | Biolegend | 399905 | B342300 |
| CD1D | anti-human CD1d | 51.1 | TCGAGTCGCTTATCA | 210 µl | Biolegend | 399905 | B342300 |
| ***Gene Name*** | ***Description*** | ***Clone*** | ***Barcode*** | ***Dilution*** | ***Source*** | ***Cat. No*** | ***Lot No.*** |
| KLRK1 | anti-human CD314 (NKG2D) | 1D11 | CGTGTTTGTTCCTCA | 210 µl | Biolegend | 399905 | B342300 |
| CR1 | anti-human CD35 | E11 | ACTTCCGTCGATCTT | 210 µl | Biolegend | 399905 | B342300 |
| B3GAT1 | anti-human CD57 Recombinant | QA17A04 | AACTCCCTATGGAGG | 210 µl | Biolegend | 399905 | B342300 |
| BTLA | anti-human CD272 (BTLA) | MIH26 | GTTATTGGACTAAGG | 210 µl | Biolegend | 399905 | B342300 |
| ICOS | anti-human/mouse/rat CD278 (ICOS) | C398.4A | CGCGCACCCATTAAA | 210 µl | Biolegend | 399905 | B342300 |
| CD58 | anti-human CD58 (LFA-3) | TS2/9 | GTTCCTATGGACGAC | 210 µl | Biolegend | 399905 | B342300 |
| ENTPD1 | anti-human CD39 | A1 | TTACCTGGTATCCGT | 210 µl | Biolegend | 399905 | B342300 |
| CX3CR1 | anti-human CX3CR1 | K0124E1 | AGTATCGTCTCTGGG | 210 µl | Biolegend | 399905 | B342300 |
| CD24 | anti-human CD24 | ML5 | AGATTCCTTCGTGTT | 210 µl | Biolegend | 399905 | B342300 |
| CR2 | anti-human CD21 | Bu32 | AACCTAGTAGTTCGG | 210 µl | Biolegend | 399905 | B342300 |
| ITGAL | anti-human CD11a | TS2/4 | TATATCCTTGTGAGC | 210 µl | Biolegend | 399905 | B342300 |
| CD79B | anti-human CD79b (Igβ) | CB3-1 | ATTCTTCAACCGAAG | 210 µl | Biolegend | 399905 | B342300 |
| CD244 | anti-human CD244 (2B4) | C1.7 | TCGCTTGGATGGTAG | 210 µl | Biolegend | 399905 | B342300 |
| SIGLEC1 | anti-human CD169 (Sialoadhesin, Siglec-1) | 7-239 | TACTCAGCGTGTTTG | 210 µl | Biolegend | 399905 | B342300 |
| ITGB7 | anti-human/mouse integrin β7 | FIB504 | TCCTTGGATGTACCG | 210 µl | Biolegend | 399905 | B342300 |
| TNFRSF13C | anti-human CD268 (BAFF-R) | 11C1 | CGAAGTCGATCCGTA | 210 µl | Biolegend | 399905 | B342300 |
| GP1BB | anti-human CD42b | HIP1 | TCCTAGTACCGAAGT | 210 µl | Biolegend | 399905 | B342300 |
| ICAM1 | anti-human CD54 | HA58 | CTGATAGACTTGAGT | 210 µl | Biolegend | 399905 | B342300 |
| SELP | anti-human CD62P (P-Selectin) | AK4 | CCTTCCGTATCCCTT | 210 µl | Biolegend | 399905 | B342300 |
| IFNGR1 | anti-human CD119 (IFN-γ R α chain) | GIR-208 | TGTGTATTCCCTTGT | 210 µl | Biolegend | 399905 | B342300 |
| TCR alpha/beta | anti-human TCR α/β | IP26 | CGTAACGTAGAGCGA | 210 µl | Biolegend | 399905 | B342300 |
| isotype | Rat IgG1, κ isotype Ctrl | RTK2071 | ATCAGATGCCCTCAT | 210 µl | Biolegend | 399905 | B342300 |
| Isotype | Rat IgG2a, κ Isotype Ctrl | RTK2758 | AAGTCAGGTTCGTTT | 210 µl | Biolegend | 399905 | B342300 |
| isotype | Armenian Hamster IgG Isotype Ctrl | HTK888 | CCTGTCATTAAGACT | 210 µl | Biolegend | 399905 | B342300 |
| IL2RB | anti-human CD122 (IL-2Rβ) | TU27 | TCATTTCCTCCGATT | 210 µl | Biolegend | 399905 | B342300 |
| TNFRSF13B | anti-human CD267 (TACI) | 1A1 | AGTGATGGAGCGAAC | 210 µl | Biolegend | 399905 | B342300 |
| FCER1A | anti-human FcεRIα | AER-37 (CRA-1) | CTCGTTTCCGTATCG | 210 µl | Biolegend | 399905 | B342300 |
| ITGA2B | anti-human CD41 | HIP8 | ACGTTGTGGCCTTGT | 210 µl | Biolegend | 399905 | B342300 |
| TNFRSF9 | anti-human CD137 (4-1BB) | 4B4-1 | CAGTAAGTTCGGGAC | 210 µl | Biolegend | 399905 | B342300 |
| CD163 | anti-human CD163 | GHI/61 | GCTTCTCCTTCCTTA | 210 µl | Biolegend | 399905 | B342300 |
| CD83 | anti-human CD83 | HB15e | CCACTCATTTCCGGT | 210 µl | Biolegend | 399905 | B342300 |
| IL4R | anti-human CD124 (IL-4Rα) | G077F6 | CCGTCCTGATAGATG | 210 µl | Biolegend | 399905 | B342300 |
| ***Gene Name*** | ***Description*** | ***Clone*** | ***Barcode*** | ***Dilution*** | ***Source*** | ***Cat. No*** | ***Lot No.*** |
| ANPEP | anti-human CD13 | WM15 | TTTCAACGCCCTTTC | 210 µl | Biolegend | 399905 | B342300 |
| CD2 | anti-human CD2 | TS1/8 | TACGATTTGTCAGGG | 210 µl | Biolegend | 399905 | B342300 |
| CD226 | anti-human CD226 (DNAM-1) | 11A8 | TCTCAGTGTTTGTGG | 210 µl | Biolegend | 399905 | B342300 |
| ITGB1 | anti-human CD29 | TS2/16 | GTATTCCCTCAGTCA | 210 µl | Biolegend | 399905 | B342300 |
| CLEC4C | anti-human CD303 (BDCA-2) | 201A | GAGATGTCCGAATTT | 210 µl | Biolegend | 399905 | B342300 |
| ITGA2 | anti-human CD49b | P1E6-C5 | GCTTTCTTCAGTATG | 210 µl | Biolegend | 399905 | B342300 |
| CD81 | anti-human CD81 (TAPA-1) | 5A6 | GTATCCTTCCTTGGC | 210 µl | Biolegend | 399905 | B342300 |
| IGHD | anti-human IgD | IA6-2 | CAGTCTCCGTAGAGT | 210 µl | Biolegend | 399905 | B342300 |
| ITGB2 | anti-human CD18 | TS1/18 | TATTGGGACACTTCT | 210 µl | Biolegend | 399905 | B342300 |
| CD28 | anti-human CD28 | CD28.2 | TGAGAACGACCCTAA | 210 µl | Biolegend | 399905 | B342300 |
| CD38 | anti-human CD38 | HIT2 | TGTACCCGCTTGTGA | 210 µl | Biolegend | 399905 | B342300 |
| IL7R | anti-human CD127 (IL-7Rα) | A019D5 | GTGTGTTGTCCTATG | 210 µl | Biolegend | 399905 | B342300 |
| PTPRC | anti-human CD45 | HI30 | TGCAATTACCCGGAT | 210 µl | Biolegend | 399905 | B342300 |
| CD22 | anti-human CD22 | S-HCL-1 | GGGTTGTTGTCTTTG | 210 µl | Biolegend | 399905 | B342300 |
| TFRC | anti-human CD71 | CY1G4 | CCGTGTTCCTCATTA | 210 µl | Biolegend | 399905 | B342300 |
| DPP4 | anti-human CD26 | BA5b | GGTGGCTAGATAATG | 210 µl | Biolegend | 399905 | B342300 |
| CD36 | anti-human CD36 | 5-271 | TTCTTTGCCTTGCCA | 210 µl | Biolegend | 399905 | B342300 |
| KIR2DL1 | anti-human CD158 (KIR2DL1/S1/S3/S5) | HP-MA4 | TATCAACCAACGCTT | 210 µl | Biolegend | 399905 | B342300 |
| ITGA1 | anti-human CD49a | TS2/7 | ACTGATGGACTCAGA | 210 µl | Biolegend | 399905 | B342300 |
| ITGA4 | anti-human CD49d | 9F10 | CCATTCAACTTCCGG | 210 µl | Biolegend | 399905 | B342300 |
| NT5E | anti-human CD73 (Ecto-5'-nucleotidase) | AD2 | CAGTTCCTCAGTTCG | 210 µl | Biolegend | 399905 | B342300 |
|  | anti-human TCR Vα7.2 | 3C10 | TACGAGCAGTATTCA | 210 µl | Biolegend | 399905 | B342300 |
|  | anti-human TCR Vδ2 | B6 | TCAGTCAGATGGTAT | 210 µl | Biolegend | 399905 | B342300 |
| OLR1 | anti-human LOX-1 | 15C4 | ACCCTTTACCGAATA | 210 µl | Biolegend | 399905 | B342300 |
| KIR2DL3 | anti-human CD158b (KIR2DL2/L3, NKAT2) | DX27 | GACCCGTAGTTTGAT | 210 µl | Biolegend | 399905 | B342300 |
| KIR3DL1 | anti-human CD158e1 (KIR3DL1, NKB1) | DX9 | GGACGCTTTCCTTGA | 210 µl | Biolegend | 399905 | B342300 |
| SLAMF7 | anti-human CD319 (CRACC) | 162.1 | AGTATGCCATGTCTT | 210 µl | Biolegend | 399905 | B342300 |
| CD99 | anti-human CD99 | 3B2/TA8 | ACCCGTCCCTAAGAA | 210 µl | Biolegend | 399905 | B342300 |
| CLEC12A | anti-human CLEC12A | 50C1 | CATTAGAGTCTGCCA | 210 µl | Biolegend | 399905 | B342300 |
| SLAMF6 | anti-human CD352 (NTB-A) | NT-7 | AGTTTCCACTCAGGC | 210 µl | Biolegend | 399905 | B342300 |
| KLRD1 | anti-human CD94 | DX22 | CTTTCCGGTCCTACA | 210 µl | Biolegend | 399905 | B342300 |
| IGKC | anti-human Ig light chain κ | MHK-49 | AGCTCAGCCAGTATG | 210 µl | Biolegend | 399905 | B342300 |
| ***Gene Name*** | ***Description*** | ***Clone*** | ***Barcode*** | ***Dilution*** | ***Source*** | ***Cat. No*** | ***Lot No.*** |
| LILRB1 | anti-human CD85j (ILT2) | GHI/75 | CCTTGTGAGGCTATG | 210 µl | Biolegend | 399905 | B342300 |
| FCER2 | anti-human CD23 | EBVCS-5 | TCTGTATAACCGTCT | 210 µl | Biolegend | 399905 | B342300 |
|  | anti-human Ig light chain λ | MHL-38 | CAGCCAGTAAGTCAC | 210 µl | Biolegend | 399905 | B342300 |
| SIGLEC7 | anti-human CD328 (Siglec-7) | 6-434 | CTTAGCATTTCACTG | 210 µl | Biolegend | 399905 | B342300 |
| ADGRG1 | anti-human GPR56 | CG4 | GCCTAGTTTCCGTTT | 210 µl | Biolegend | 399905 | B342300 |
| HLA-E | anti-human HLA-E | 3D12 | GAGTCGAGAAATCAT | 210 µl | Biolegend | 399905 | B342300 |
| CD82 | anti-human CD82 | ASL-24 | TCCCACTTCCGCTTT | 210 µl | Biolegend | 399905 | B342300 |
| CD101 | anti-human CD101 (BB27) | BB27 | CTACTTCCCTGTCAA | 210 µl | Biolegend | 399905 | B342300 |
| C5AR1 | anti-human CD88 (C5aR) | S5/1 | GCCGCATGAGAAACA | 210 µl | Biolegend | 399905 | B342300 |
| GGT1 | anti-human CD224 | KF29 | CTGATGAGATGTCAG | 210 µl | Biolegend | 399905 | B342300 |
| ***Additional Spike-In Antibodies for BAL Measurements*** | | | | | | | |
| CD206 | anti-human CD206 | 15-2 | TCAGAACGTCTAACT | 1:250 | Biolegend | 321147 | B318122 |
| CD298 | anti-human CD298 (PD1) | EH12.2H7 | ACAGCGCCGTATTTA | 1:250 | Biolegend | 329963 | B334416 |
| ***Additional Spike-In Antibodies for PBMC Measurements*** | | | | | | | |
| CCR2 | Anti-human CCR2 | K036C2 | GAGTTCCCTTACCTG | 1:50 | Biolegend | 357235 | B334431 |
| Hashtag 9 | Hashtag 9 | LNH-94;2M2 | CAGTAGTCACGGTCA | 1:50 | Biolegend | 394677 | B334825 |
| Hashtag 13 | Hashtag 13 | LNH-94; 2M2 | AAATCTCTCAGGCTC | 1:50 | Biolegend | 394685 | B368273 |

**Table S4. Quality Control Metrics of CITE-seq Data**

| ***Sample*** | ***Number of Cells*** | ***Number of Cells Passing QC*** | ***% Cells Passing QC*** |
| --- | --- | --- | --- |
| Participant 1: B1 – BAL | 5067 | 3718 | 73% |
| Participant 2: B1 – BAL | 6063 | 5009 | 83% |
| Participant 2: B2 – BAL | 5706 | 4809 | 84% |
| Participant 3: B1 – BAL | 7419 | 6037 | 81% |
| Participant 4: B1 – BAL | 5985 | 3712 | 62% |
| Participant 4: B2 – BAL | 9042 | 7593 | 84% |
| Participant 5: B1 – BAL | 6150 | 4797 | 79% |
| Participant 6: B1 – BAL | 7356 | 5367 | 73% |
| Participant 7: B1 – BAL | 7832 | 6839 | 87% |
| Participant 7: B2 – BAL | 7441 | 6215 | 84% |
| Participant 8: B1 – BAL | 5700 | 4948 | 87% |
| Participant 8: B2 – BAL | 6022 | 5273 | 88% |
| ***BAL Samples: Median (IQR)*** | | ***5,141 (4,800 – 6,171)*** | ***84% (75% - 86%)*** |
| Participant 1: B1 – PBMC | 9216 | 7373 | 80% |
| Participant 2: B1 – PBMC | 7395 | 6667 | 90% |
| Participant 2: B2 – PBMC | 8147 | 7047 | 86% |
| Participant 3: B1 – PBMC | 14784 | 13897 | 94% |
| Participant 4: B1 – PBMC | 10440 | 10171 | 97% |
| Participant 4: B2 – PBMC | 8990 | 8731 | 97% |
| Participant 5: B1 – PBMC | 11376 | 11216 | 98% |
| Participant 6: B1 – PBMC* | 11680 | 10766 | 92% |
| Participant 7: B1 – PBMC | 8909 | 8491 | 95% |
| Participant 7: B2 – PBMC | 3553 | 3387 | 95% |
| Participant 8: B1 – PBMC | 6748 | 6387 | 95% |
| Participant 8: B2 – PBMC | 8160 | 7733 | 95% |
| ***PBMC Samples: Median (IQR)*** | | ***8,112 (6,762 – 10,617)*** | ***95% (91% - 97%)*** |

B1 = Bronchoscopy 1; B2 = Bronchoscopy 2; BAL = bronchoalveolar lavage; PBMC = peripheral blood mononuclear cell

* Participant 6’s PBMCs were collected 3 days following their B1 BAL collection. All other PBMC samples were collected at the same time as the BAL collection.

Quality control (QC) thresholds excluded cells that had < 500 genes, > 4,500 genes, or had > 12.5% of reads mapping to mitochondrial genes.

**Table S5. Top 20 Marker Genes for Each Alveolar Myeloid Cluster**

| ***Cluster 0***  ***“CD163/LGMN”*** | ***Cluster 1***  ***“FCN1”*** | ***Cluster 2***  ***“Intermediate”*** | ***Cluster 3***  ***“Mature”*** | ***Cluster 4***  ***“Inflammatory”*** | ***Cluster 5***  ***“IFN-Related”*** | ***Cluster 6***  ***“Matricellular”*** | ***Cluster 7***  ***“DCs”*** | ***Cluster 8***  ***“Metallothionein”*** |
| --- | --- | --- | --- | --- | --- | --- | --- | --- |
| *RNASE1* | *FCN1* | *APOC1* | *FABP4* | *S100A12* | *CXCL10* | *SPP1* | *CCL17* | *MT1G* |
| *LGMN* | *RETN* | *GCHFR* | *IFI27* | *S100A9* | *CCL8* | *CHIT1* | *CD1C* | *MT2A* |
| *CCL13* | *LYZ* | *ACP5* | *SERPING1* | *S100A8* | *ISG15* | *LPL* | *WFDC21P* | *MT1X* |
| *HMOX1* | *CORO1A* | *FBP1* | *C1QB* | *IL1B* | *IFIT2* | *CD9* | *S100B* | *MT1H* |
| *CCL2* | *LST1* | *APOE* | *RBP4* | *CCL3L3* | *MX1* | *CHI3L1* | *CST7* | *MT1F* |
| *CTSL* | *VSIR* | *VSIG4* | *CCL18* | *G0S2* | *IFIT1* | *MMP7* | *HLA-DQA1* | *MT1E* |
| *PLTP* | *VCAN* | *C1QA* | *C1QA* | *CXCL8* | *IFIT3* | *GSN* | *RGS1* | *MT1M* |
| *SPP1* | *AIF1* | *AGRP* | *CES1* | *CCL3* | *RSAD2* | *TREM2* | *IFITM1* | *MT1L* |
| *CD163* | *FOS* | *CYP27A1* | *NUPR1* | *TIMP1* | *TNFSF10* | *LIPA* | *NAPSB* | *TPT1* |
| *FOLR2* | *ASGR1* | *MARCO* | *C1QC* | *CCL4L2* | *IFITM1* | *MATK* | *HLA-DPB1* | *RPS26* |
| *MARCKS* | *LSP1* | *LTA4H* | *FN1* | *CCL4* | *IFITM3* | *LINC02345* | *GPR183* | *MIF* |
| *STAB1* | *ZFP36* | *TREM2* | *AC026369.3* | *SOD2* | *GBP1* | *FABP3* | *HLA-DQB1* | *RGCC* |
| *CTSB* | *S100A4* | *AC009093.3* | *LGALS3BP* | *EREG* | *HERC5* | *FBP1* | *HLA-DPA1* | *OLR1* |
| *F13A1* | *RPL35A* | *ALDH2* | *AC009093.3* | *NLRP3* | *RNF213* | *SPARC* | *CD1E* | *THBS1* |
| *CTSD* | *RPS24* | *C1QC* | *INHBA* | *PTGS2* | *MX2* | *CAMK1* | *PPP1R14A* | *HCST* |
| *HSPA5* | *RPS3A* | *C1QB* | *APOC1* | *SERPINB2* | *APOBEC3A* | *SDC2* | *FCER1A* | *IL1B* |
| *GLUL* | *RPL41* | *UBB* | *PHLDA3* | *CCL7* | *EPSTI1* | *A2M* | *CLEC10A* | *RPL13P12* |
| *TGFBI* | *CCR2* | *MGST3* | *PDLIM1* | *NFKBIA* | *TNFSF13B* | *GPNMB* | *ARL4C* | *BTG1* |
| *PLA2G7* | *RPL18* | *CD68* | *TFRC (CD71)* | *SRGN* | *IRF7* | *PLA2G7* | *CD74* | *ACP5* |
| *CALR* | *ARHGDIB* | *GRN* | *ALDH2* | *CD300E* | *OAS1* | *MFSD12* | *PKIB* | *ZFP36* |

**Table S6. Summary of Published Datasets Compared with Current Dataset**

| ***Myeloid Cluster Name (Cell-Surface Protein)*** | ***Marker Genes*** | ***Cell-Surface Proteins*** | ***Tissue/Disease*** | ***Literature/Population*** |
| --- | --- | --- | --- | --- |
| ***CD163/LGMN Macrophages***  ***(CD163)*** | *RNASE1*  *LGMN*  *HMOX1* | CD163 | Explant and BAL/COVID-19 | “CD163+ Macrophages” ^1^ |
|  |  |  | Explant/Non-diseased | “CD163++” ^2^ |
|  |  |  | Explant/IPF | “CD163” ^3^ |
| ***FCN1 Monocytes***  ***(LOX-1)*** | *FCN1*  *RETN*  *LYZ*  *LST1* | Not previously defined in humans | BAL/COVID-19 | “FCN1^hi^” ^4^ |
|  |  |  | Explant and BAL/COVID-19 | “FCN1” ^1^ |
|  |  |  | Explant/IPF | “FCN1^HI^” ^5^ |
|  |  |  | BAL/COVID-19 | “monocyte-FCN1” ^6^ |
| ***Intermediate Monocyte-Macrophages***  ***(CD123)*** | *FBP1*  *APOE*  *APOC1*  *GCHFR* | Not previously defined in humans | Not previously defined | Not previously defined in humans |
| ***Mature Macrophages***  ***(CD71)*** | *FABP4*  *IFI27*  *SERPING1*  *C1QB*  *RBP4* | CD206  CD169  CD71  CD274 (PD-L1)  CD14^LO^  CD11b  CD141 | BAL/Healthy | “M0 – M2, M4, M6, M8 – M9, M11” ^7^ |
|  |  |  | BAL/Healthy/CF | “AMs.S1” ^8^ |
|  |  |  | BAL/Healthy | “AMs” ^9,10^ |
|  |  |  | BAL/Healthy/ARDS | “CD169^HI^PD-L1^HI^” ^11^ |
|  |  |  | BAL/Healthy | “CD71^HI^” ^12^ |
|  |  |  | Explant/Non-diseased | “AMs” ^2,13^ |
|  |  |  | BAL/Allergy | “Mac-1” ^14^ |
| ***Inflammatory Monocytes***  ***(CD35)*** | *S100A12*  *S100A9*  *S100A8*  *IL1B*  *CCL3L3* | CD14  CD35 | BAL/ARDS | “alveolar monocyte” ^15^ |
|  |  |  | BAL/Healthy | “m5” ^7^ |
|  |  |  | BAL/COVID-19 | “monocyte-IL-1b” ^6^ |
|  |  |  | Explant/Non-Diseased | “monocytes” ^16^ |
| ***IFN-Related Macrophages***  ***(CD48)*** | *CCL8*  *ISG15*  *IFIT2*  *MX1* | Not previously defined in humans | BAL/COVID-19 | “group1, group2” ^4^ |
|  |  |  | BAL/COVID-19 | “Module 15” ^17^ |
|  |  |  | Explant and BAL/COVID-19 | “AM2 ISG-antiviral” ^1^ |
|  |  |  | BAL/Allergy | “MC1” ^14^ |
|  |  |  | BAL/Healthy/CF | “IFN.AMs” ^8^ |
| ***Matricellular Macrophages***  ***(CD86)*** | *SPP1*  *CHIT1*  *CD9*  *CHI3L1* | Not previously defined in humans | Explant/IPF | “Pro-fibrotic monocyte derived AMs” ^18^ |
|  |  |  | BAL/COVID-19 | “alternative M2-like” ^4^ |
|  |  |  | BAL/Healthy | “mo2” ^7^ |
|  |  |  | BAL/Allergy | “MC2” ^14^ |
|  |  |  | Explant/IPF | “SPP1^HI^” ^5^ |
| ***Dendritic Cells***  ***(CD1c)*** | *CCL17*  *CDC1* | CD1c | BAL/Healthy | “cDC2” ^7^ |
|  |  |  | BAL/COVID-19 | “mDCs” ^4^ |
|  |  |  | Explant/Non-diseased | “DCs” ^13^ |
| ***Metallothionein Macrophages***  ***(CD44)*** | *MT1G*  *MT2A*  *MT1X*  *MT1F* | Not previously defined in humans | BAL/Healthy | “m6” ^7^ |
|  |  |  | BAL/Healthy | “MIP-1a-expressing” ^19^ |
|  |  |  | BAL/Healthy/CF | “MT.AMs” ^8^ |

**Table S7. Characteristics of Participants with or without ARDS**

|  | ***No ARDS***  ***(n = 4)*** | ***ARDS***  ***(n = 4)*** |
| --- | --- | --- |
| **Intubation-to-BAL Interval (days)** | 4, 2 – 5 | 4, 3 – 5 |
| **P/F Ratio** | 324, 183 – 440 | 198, 119 – 234 |
| **OI** | 3.2, 2.7 – 8.8 | 6.6, 4.7 – 10.2 |
| **% PMNs** | 33, 1 – 61 | 72, 66 – 82 |
| **% AMs** | 56, 36 – 89 | 23, 13 – 29 |
| **SOFA** | 8, 4 – 10 | 9, 7 - 12 |

AM = alveolar macrophage; ARDS = acute respiratory distress syndrome; adjudicated at time of bronchoscopy; P/F ratio = P_a_O_2_/F_i_O_2_ ratio; calculated at the time of bronchoscopy (if missing arterial blood gas, SaO2/FiO2 ratio is reported); OI = oxygenation index; PMN = polymorphonuclear leukocyte; Sample Day = interval of days between initiation of invasive mechanical ventilation and bronchoscopy; SOFA = sequential organ failure assessment; calculated at time of bronchoscopy

**Table S8. Top 20 Marker Genes for “Integrated” Blood-Lung Clusters**

| ***Cluster 0*** | ***Cluster 1*** | ***Cluster 2*** | ***Cluster 3*** | ***Cluster 4*** | ***Cluster 5*** | ***Cluster 6*** |
| --- | --- | --- | --- | --- | --- | --- |
| *S100A8* | *APOE* | *CCL4* | *ISG15* | *CDKN1C* | *CCL17* | *MALAT1* |
| *S100A12* | *APOC1* | *CCL4L2* | *MX1* | *RHOC* | *HLA-DQA1* | *GPX1P1* |
| *S100A9* | *ACP5* | *FABP4* | *LY6E* | *IFITM1* | *SPP1* | *TMEM176B* |
| *ATP5F1E* | *SPP1* | *CCL3L3* | *IFI6* | *FCGR3A (CD16)* | *HLA-DPB1* | *PLTP* |
| *MTRNR2L12* | *TREM2* | *IFI27* | *NCF1* | *IFITM2* | *HLA-DPA1* | *RPL13P12* |
| *G0S2* | *RNASE1* | *CCL18* | *IFITM3* | *IFITM3* | *S100B* | *F13A1* |
| *ATP5MG* | *GCHFR* | *CXCL3* | *APOBEC3A* | *IFI30* | *HLA-DQB1* | *NEAT1* |
| *ATP5MC2* | *FBP1* | *CCL3* | *IFI44* | *LST1* | *IL7R* | *MT-RNR2* |
| *SLC2A3* | *CHIT1* | *IL1B* | *IFIT2* | *LILRB1* | *MALAT1* | *FOLR2* |
| *RPL36A* | *MRC1* | *C1QB* | *IFIT1* | *MTSS1* | *HLA-DRA* | *CSF1R* |
| *C1QB* | *S100A12* | *ATP5F1E* | *APOC1* | *MT-RNR2* | *S100A12* | *ATP5F1E* |
| *APOC1* | *S100A8* | *MTRNR2L12* | *APOE* | *APOC1* | *S100A8* | *MTRNR2L12* |
| *APOE* | *S100A9* | *IFI30* | *C1QB* | *MT-RNR1* | *S100A9* | *IFI30* |
| *CCL18* | *ATP5F1E* | *ATP5MC2* | *C1QA* | *APOE* | *ATP5F1E* | *ATP5MC2* |
| *C1QA* | *MTRNR2L12* | *ATP5MG* | *HLA-DQB1* | *CCL2* | *FCN1* | *CRIP1* |
| *C1QC* | *IFI30* | *S100A12* | *C1QC* | *MALAT1* | *MTRNR2L12* | *ATP5MG* |
| *FABP4* | *ATP5MC2* | *S100A8* | *HLA-DRB5* | *GPX1* | *NCF1* | *RPL36A* |
| *ACP5* | *ATP5MG* | *S100A9* | *HLA-DRB1* | *CCL18* | *IFI30* | *EEF1G* |
| *FN1* | *G0S2* | *RPL36A* | *HLA-DQA1* | *ATP5E* | *CRIP1* | *RPL17* |
| *MARCO* | *NCF1* | *NCF1* | *HLA-DPA1* | *S100A12* | *CXCL8* | *EIF4A1* |

**Table S9. Most Discriminatory Cell-Surface Proteins Between Alveolar Monocyte and Macrophage Clusters**

| ***Cluster 0***  ***“CD163/LGMN”*** | ***Cluster 1***  ***“FCN1”*** | ***Cluster 2***  ***“Intermediate”*** | ***Cluster 3***  ***“Mature”*** | ***Cluster 4***  ***“Inflammatory”*** | ***Cluster 5***  ***“IFN-Related”*** | ***Cluster 6***  ***“Matricellular”*** | ***Cluster 7***  ***“DCs”*** | ***Cluster 8***  ***“Metallothionein”*** |
| --- | --- | --- | --- | --- | --- | --- | --- | --- |
| CD163 | LOX-1 | CD123 | CD71 | CD35 | CD48 | CD86 | CD1c | CD44 |
| CD352 | CD48 | CD169 | CD169 | CD48 | CD69 | CD49f | FceRIa | CD11c |
| CD47 | CLEC12A | CD49f | CD103 | CD14 | CD71 | CD11a | CD39 | CLEC12A |
| CD40 | CD69 | CD16 | CD107a | CD244 | CD49f | CD38 | CD2 | CD32b |
| HLA-DR | CD244 | CD206 | CD274 (PD-L1) | CD36 | CD14 | CD35 | CD226 | CD11b |

Supplementary Data 1 provides a sortable list of the normalized expression for all cell-surface proteins per each transcriptional cluster

**Table S10. Flow Cytometry Cell-Surface Antibody Panel**

| ***Cell Surface Protein*** | ***Clone*** | ***Conjugated***  ***Fluorophore*** | ***Manufacturer*** | ***Catalog Number*** |
| --- | --- | --- | --- | --- |
| CD45 | 2D1 | Alexa Fluor 700 | BioLegend | 368513 |
| CD3 | UCHT1 | Brilliant Violet 570 | BioLegend | 300436 |
| CD11b | ICRF44 | APC | BioLegend | 301309 |
| CD14 | M5E2 | APC/Cyanine7 | BioLegend | 301819 |
| CD15 | HI98 | Brilliant Ultra Violet 395 | BD Biosciences | 563872 |
| CD71 | CY1G4 | Brilliant Violet 650 | BioLegend | 334115 |
| CD163 | GHI/61 | Brilliant Violet 605 | BioLegend | 333616 |
| CD172a | 15-414 | FITC | BioLegend | 372107 |
| CD192 | K036C2 | PE/Dazzle 594 | BioLegend | 357221 |
| CD169 | 7-239 | Brilliant Violet 421 | BioLegend | 346017 |
| CD206 | 15-2 | Brilliant Violet 785 | BioLegend | 321142 |
| CD274 | 29E.2A3 | Brilliant Violet 711 | BioLegend | 329722 |
| CD282 | TL2.1 | PE | BioLegend | 309707 |
| CD326 | 9C4 | PE/Cyanine7 | BioLegend | 324221 |

**Figure S1. Clinical and Biomarker Trajectories of Enrolled Participants**


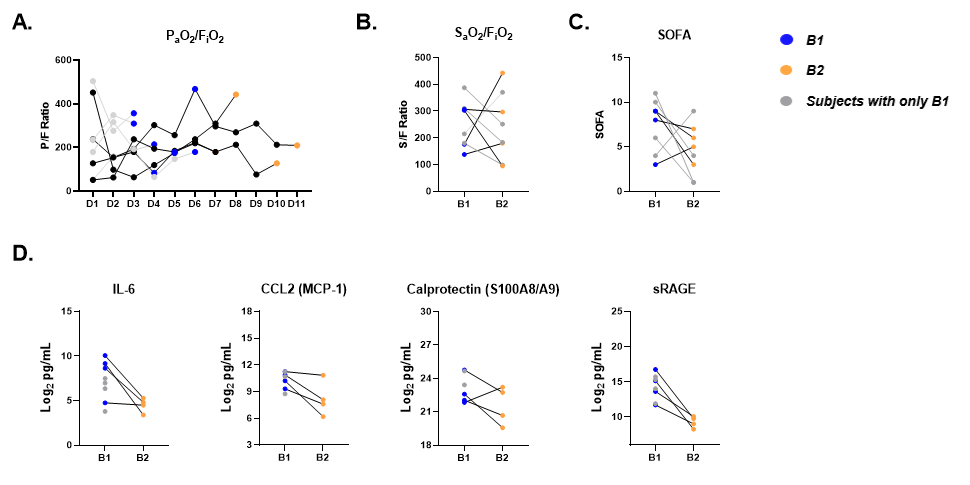


**Figure S1. Clinical and Biomarker Trajectories of Enrolled Participants.** Participants were intubated and started on mechanical ventilation at Day 1 (D1). We performed serial research bronchoscopies at two timepoints (B1 or B2). Four participants had both a B1 and B2 (eight samples) and four participants had only a B1 (four samples). **(A)** Trajectory of P_a_O_2_/F_i_O_2_ ratio (P/F). Each dot is the lowest P/F value for the day and lines connect serial measurements from an individual patient. Gray color represents participants who were only sampled at B1. **(B)** Trajectory of S_a_O_2_/F_i_O_2_ ratio. If a participant did not undergo a B2 due to extubation, their S_a_O_2_/F_i_O_2_ 4 days after B1 is shown. **(C)** Trajectory of sequential organ failure assessment (SOFA) scores. If a participant did not undergo a B2 due to extubation, their SOFA 4 days after B1 is shown. **(D)** Trajectory of BALF biomarker levels. Lines connect serial measurements from an individual patient.

**Figure S2. Distribution of the Number of Genes and Cells Detected for Each Participant**


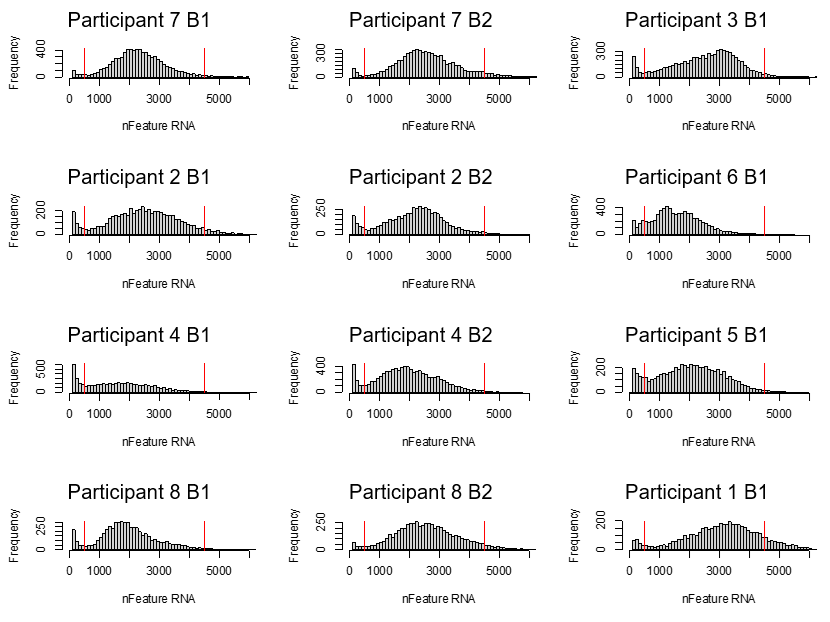


**Figure S2. Distribution of the Number of Genes per Cell Detected for Each Participant.** Histograms displaying the distribution of genes (x-axis) per cell (y-axis) for each sample. We excluded cells with < 500 or > 4,500 genes (designated with red line).

**Figure S3. Distribution of Percentage of Reads Mapping to Mitochondrial Genes per Cell for Each Participant**


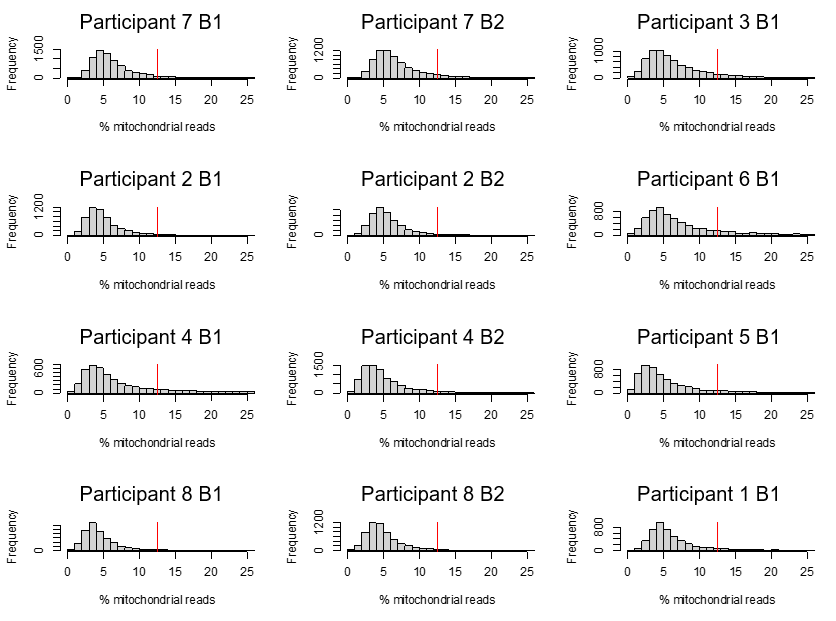


**Figure S3. Distribution of Percentage of Reads Mapping to Mitochondrial Genes per Cell for Each Participant.** Histograms displaying the % of reads mapping to mitochondrial genes (x-axis) per cell (y-axis) for each sample. We excluded cells with > 12.5% of reads mapping to mitochondrial genes (designated with red line). The two most differentially expressed genes in the cells with > 12.5% of reads mapping to mitochondrial reads vs. cells with < 12.5% of reads mapping to mitochondrial genes were *NEAT2* (also known as *MALAT1*) (log_2_ FC 1.43, p-value < 1 e -95) and *NEAT1* (log_2_ FC 1.33, p-value < 1 e -95. *NEAT2* and *NEAT1* (noncoding nuclear-enriched abundant transcript 1 and 2) are almost exclusively expressed in the cell nucleus (*BMC Genomics* 2007;8:39) and have been consistently observed in dead/dying cells detected in poly-A captured scRNA-seq data.

**Figure S4. Associations Between Alveolar Myeloid Subsets and Biomarker Levels**


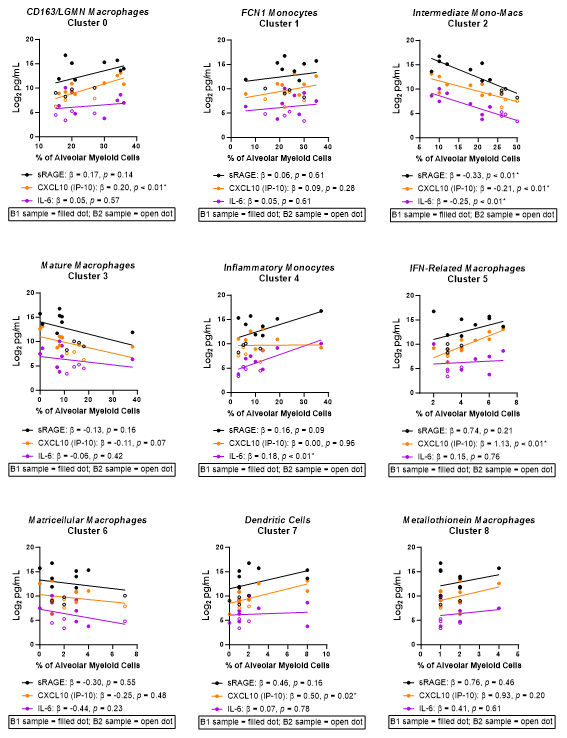


**Figure S4. Associations Between Alveolar Myeloid Subsets and Biomarker Levels.** We used linear regression to test for associations between the percentage of each alveolar myeloid subset as a proportion of all alveolar myeloid cells (x-axis) and biomarker levels measured from BAL fluid (y-axis). Depicted are the individual values and linear regression best-fit line (n = 8 unique participants at sampled at B1; n = 4 participants sampled again at B2). P-values test whether the slope (β-coefficient) is significantly non-zero. * Designates nominal P-value < 0.05 that is not adjusted for multiple hypothesis testing.

**Figure S5. Associations Between Alveolar Myeloid Subsets and Clinical Severity**


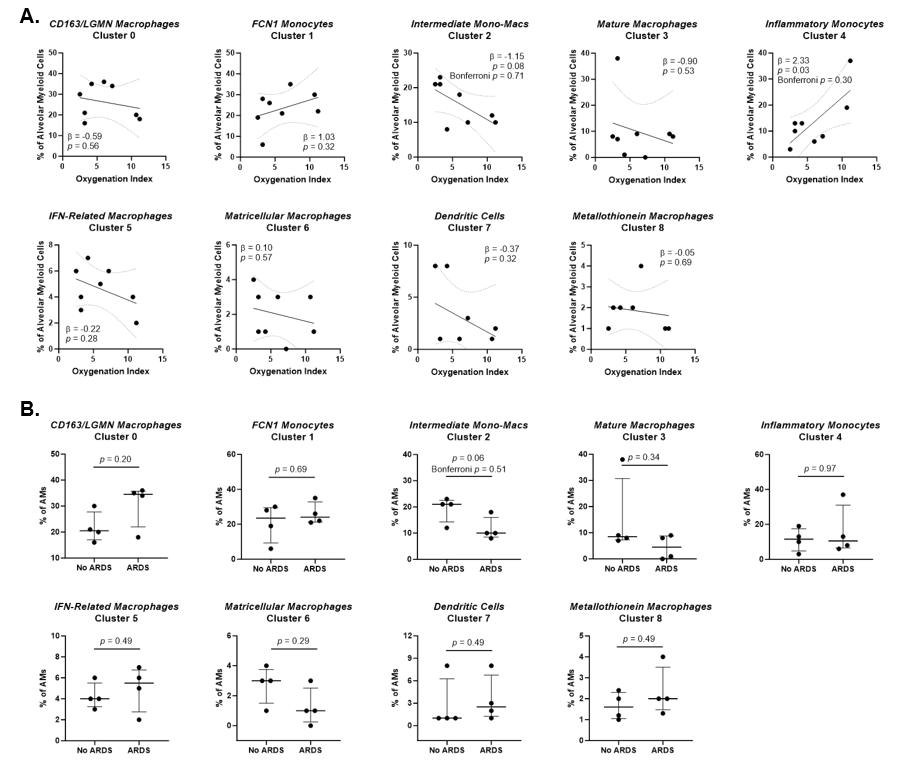


**Figure S5. Associations Between Alveolar Myeloid Subsets and Clinical Severity. (A)** Associations between the proportion of alveolar myeloid subsets as a percentage of all alveolar myeloid cells (y-axis) and oxygenation index (OI) (x-axis). OI is a measure of respiratory failure severity that accounts for both oxygenation and mean airway pressure being delivered by mechanical ventilation. Higher values indicate more severe respiratory failure. Depicted are the individual values, linear regression best-fit line, and 95% confidence intervals (n = 8 unique participants). P-values test whether the slope (β-coefficient) is significantly non-zero and are nominal. Bonferroni p-values are adjusted for 9 statistical tests (multiple hypothesis testing for an association between each of the nine subsets and the clinical outcome). **(B)** The percentage of each alveolar myeloid subset as a proportion of all alveolar myeloid cells in participants with or without ARDS. Depicted are the individual values, median, and interquartile range of each subset as a proportion of all alveolar myeloid cells (n = 8 unique participants). P-values were generated with two-sided Mann-Whitney tests and are nominal. Bonferroni p-values are adjusted for 9 statistical tests (multiple hypothesis testing for an association between each of the nine subsets and the clinical outcome).

**Figure S6. Associations Between Alveolar Myeloid Subsets and Age**


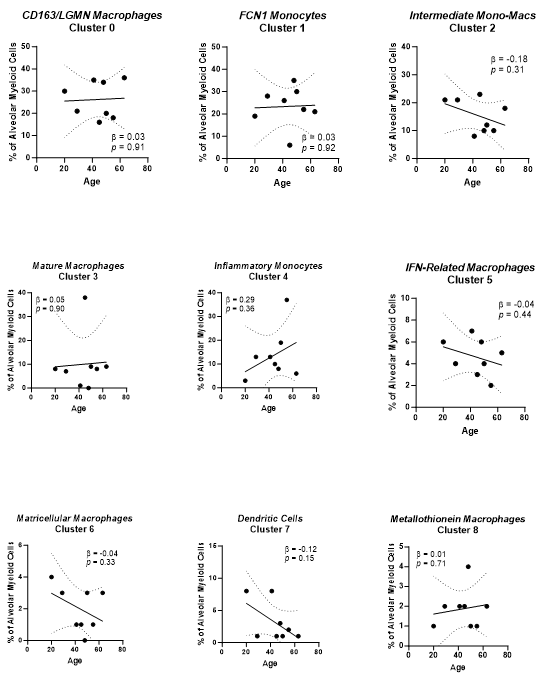


**Figure S6. Associations Between Alveolar Myeloid Subsets and Age.** We used linear regression to test for associations between age (x-axis) and the percentage of each alveolar myeloid subset as a proportion of all alveolar myeloid cells (y-axis). Depicted are the individual values, linear regression best-fit line, and 95% confidence intervals (n = 8 unique participants). P-values test whether the slope (β-coefficient) is significantly non-zero.

**Figure S7. RNA Velocity of Alveolar Monocyte and Macrophage Subsets**


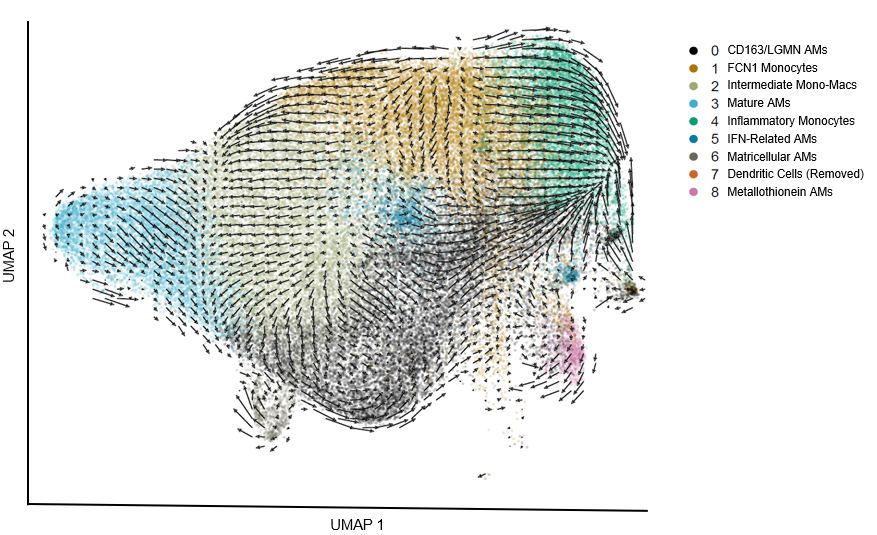


**Figure S7. RNA Velocity of Alveolar Monocyte and Macrophage Subsets.** RNA velocity field projected on the alveolar myeloid UMAP. Arrows show the local average velocity evaluated on a regular grid (alveolar macrophage = AM). Dendritic cells were removed for RNA velocity analysis.

**Figure S8. Integrated Blood-Lung Myeloid Cell Clustering**


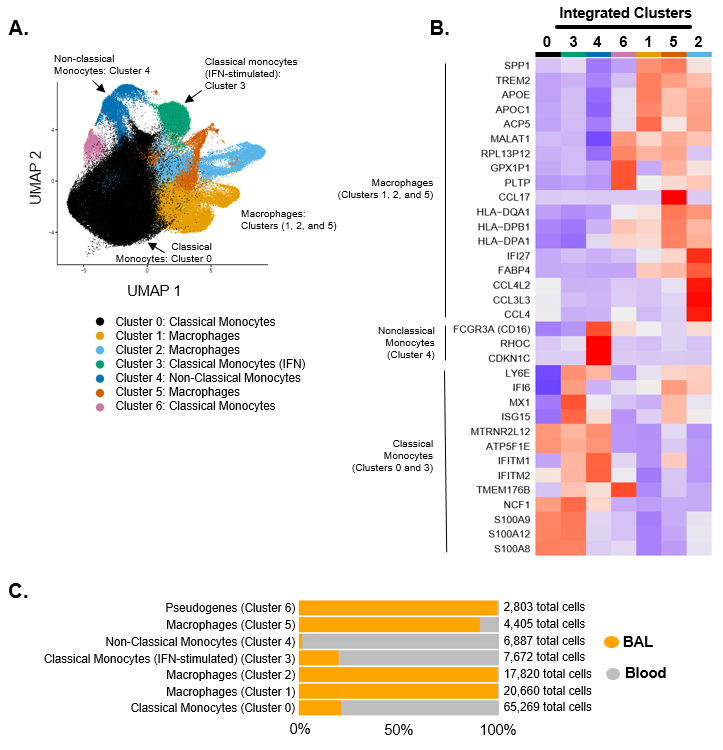


**Figure S8. Integrated Blood-Lung Myeloid Cell Clustering. (A)** Uniform manifold approximation and projection (UMAP) plot displaying clusters derived from myeloid cells (monocytes and DCs) collected from blood sampling and alveolar myeloid cells (monocytes, macrophages, and DCs) collected from BAL fluid (“integrated” blood-lung dataset). We annotated the integrated UMAP based on gene expression signatures from Panel B. **(B)** Heatmap showing the most differentially expression genes between each of the seven clusters. A full list of the most differentially expressed genes is shown in Table S8. **(C)** Percentage bar graph displays the proportion of each cluster that came from either a blood or lung (BAL) sample. Cluster 4 (non-classical monocytes with high *FCGR3A* (CD16), *RHOC*, and *CDKN1C* gene expression) was almost exclusive derived from blood samples. In contrast, Clusters 0 and 3 (classical monocytes with high *S100A8*, *S100A9*, and *S100A12* gene expression) were each derived from ~25% blood samples and ~75% BAL samples.

**Figure S9. Cell-Surface Antigen Specificity Scores**


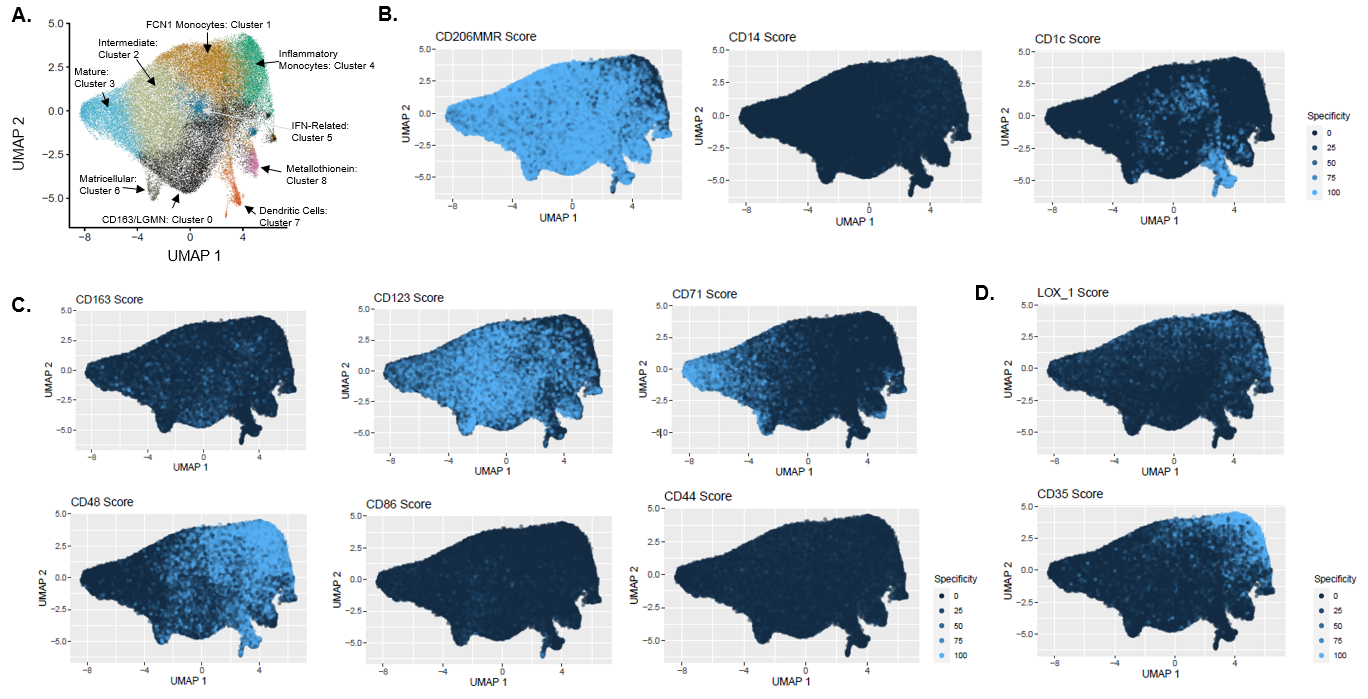


**Figure S9. Cell-Surface Protein Specificity Scores. (A)** Uniform manifold approximation and projection (UMAP) displaying the alveolar myeloid cells colored by subsets. **(B, C, and D)** Antigen-specificity scores projected onto the alveolar myeloid cell UMAP. The antigen-specificity score is the likelihood of an antigen binding to its receptor compared with the negative isotype control. **(B)** Canonical alveolar macrophage (CD206), monocyte (CD14), and dendritic cell (CD1c) cell-surface protein markers. **(C)** Most differentially expressed cell-surface proteins across the alveolar macrophage subsets. **(D)** Most differentially expressed cell-surface proteins across the alveolar monocyte subsets.

**References**

1. Wendisch, D. *et al.* SARS-CoV-2 infection triggers profibrotic macrophage responses and lung fibrosis. *Cell* **184**, 6243-6261.e27 (2021).

2. Bharat, A. *et al.* Flow Cytometry Reveals Similarities Between Lung Macrophages in Humans and Mice. *Am J Respir Cell Mol Biol* **54**, 147–149 (2016).

3. Nouno, T. *et al.* Elevation of pulmonary CD163+ and CD204+ macrophages is associated with the clinical course of idiopathic pulmonary fibrosis patients. *J Thorac Dis* **11**, 4005–4017 (2019).

4. Liao, M. *et al.* Single-cell landscape of bronchoalveolar immune cells in patients with COVID-19. *Nature Medicine* **26**, 842–844 (2020).

5. Morse, C. *et al.* Proliferating SPP1/MERTK-expressing macrophages in idiopathic pulmonary fibrosis. *Eur Respir J* **54**, 1802441 (2019).

6. Wauters, E. *et al.* Discriminating mild from critical COVID-19 by innate and adaptive immune single-cell profiling of bronchoalveolar lavages. *Cell Res* **31**, 272–290 (2021).

7. Mould, K. J. *et al.* Airspace Macrophages and Monocytes Exist in Transcriptionally Distinct Subsets in Healthy Adults. *Am J Respir Crit Care Med* **203**, 946–956 (2021).

8. Li, X. *et al.* ScRNA-seq expression of IFI27 and APOC2 identifies four alveolar macrophage superclusters in healthy BALF. *Life Sci Alliance* **5**, e202201458 (2022).

9. Yu, Y.-R. A. *et al.* Flow Cytometric Analysis of Myeloid Cells in Human Blood, Bronchoalveolar Lavage, and Lung Tissues. *Am. J. Respir. Cell Mol. Biol.* **54**, 13–24 (2016).

10. Tighe, R. M. *et al.* Improving the Quality and Reproducibility of Flow Cytometry in the Lung. An Official American Thoracic Society Workshop Report. *Am J Respir Cell Mol Biol* **61**, 150–161 (2019).

11. Morrell, E. D. *et al.* Cytometry TOF identifies alveolar macrophage subtypes in acute respiratory distress syndrome. *JCI Insight* **3**, pii: 99281 (2018).

12. Allden, S. J. *et al.* The Transferrin Receptor CD71 Delineates Functionally Distinct Airway Macrophage Subsets during Idiopathic Pulmonary Fibrosis. *Am J Respir Crit Care Med* **200**, 209–219 (2019).

13. Desch, A. N. *et al.* Flow Cytometric Analysis of Mononuclear Phagocytes in Nondiseased Human Lung and Lung-Draining Lymph Nodes. *Am. J. Respir. Crit. Care Med.* **193**, 614–626 (2016).

14. Alladina, J. *et al.* A human model of asthma exacerbation reveals transcriptional programs and cell circuits specific to allergic asthma. *Sci Immunol* **8**, eabq6352 (2023).

15. Rosseau, S. *et al.* Phenotypic characterization of alveolar monocyte recruitment in acute respiratory distress syndrome. *American Journal of Physiology - Lung Cellular and Molecular Physiology* **279**, L25–L35 (2000).

16. Leach, S. M. *et al.* Human and Mouse Transcriptome Profiling Identifies Cross-Species Homology in Pulmonary and Lymph Node Mononuclear Phagocytes. *Cell Rep* **33**, 108337 (2020).

17. Grant, R. A. *et al.* Circuits between infected macrophages and T cells in SARS-CoV-2 pneumonia. *Nature* **590**, 635–641 (2021).

18. Reyfman, P. A. *et al.* Single-Cell Transcriptomic Analysis of Human Lung Provides Insights into the Pathobiology of Pulmonary Fibrosis. *Am. J. Respir. Crit. Care Med.* **199**, 1517–1536 (2019).

19. Reyfman, P. A. *et al.* A Novel MIP-1-Expressing Macrophage Subtype in BAL Fluid from Healthy Volunteers. *Am J Respir Cell Mol Biol* **68**, 176–185 (2023).
