# Supplementary material for: The transcriptional and phenotypic characteristics that define alveolar macrophage subsets in acute hypoxemic respiratory failure
Source: Nat Commun. 2023 Nov 17;14:7443. doi: 10.1038/s41467-023-43223-0 (PMC10656558; doi:10.1038/s41467-023-43223-0)
Supplement: Supplementary file 3 — Description of Additional Supplementary Files [file 41467_2023_43223_MOESM3_ESM.pdf]

Description of Additional Supplementary Files

Title: Supplementary Data 1

Description: Sortable List of Normalized Expression for Cell-Surface Proteins per Transcriptional Cluster
