# Supplementary material for: The transcriptional and phenotypic characteristics that define alveolar macrophage subsets in acute hypoxemic respiratory failure
Source: Nat Commun. 2023 Nov 17;14:7443. doi: 10.1038/s41467-023-43223-0 (PMC10656558; doi:10.1038/s41467-023-43223-0)
Supplement: Supplementary file 5 — Reporting Summary [file 41467_2023_43223_MOESM5_ESM.pdf]

nature portfolio

Corresponding author(s): Mikacenic

Last updated by author(s): Oct 10, 2023

Reporting Summary

Nature Portfolio wishes to improve the reproducibility of the work that we publish. This form provides structure for consistency and transparency in reporting. For further information on Nature Portfolio policies, see our Editorial Policies and the Editorial Policy Checklist.

Statistics

For all statistical analyses, confirm that the following items are present in the figure legend, table legend, main text, or Methods section.

n/a	Confirmed	
☐	☒	The exact sample size (*n*) for each experimental group/condition, given as a discrete number and unit of measurement
☐	☒	A statement on whether measurements were taken from distinct samples or whether the same sample was measured repeatedly
☐	☒	The statistical test(s) used AND whether they are one- or two-sided<br>*Only common tests should be described solely by name; describe more complex techniques in the Methods section.*
☒	☐	A description of all covariates tested
☐	☒	A description of any assumptions or corrections, such as tests of normality and adjustment for multiple comparisons
☐	☒	A full description of the statistical parameters including central tendency (e.g. means) or other basic estimates (e.g. regression coefficient) AND variation (e.g. standard deviation) or associated estimates of uncertainty (e.g. confidence intervals)
☐	☒	For null hypothesis testing, the test statistic (e.g. *F*, *t*, *r*) with confidence intervals, effect sizes, degrees of freedom and *P* value noted<br>*Give P values as exact values whenever suitable.*
☒	☐	For Bayesian analysis, information on the choice of priors and Markov chain Monte Carlo settings
☒	☐	For hierarchical and complex designs, identification of the appropriate level for tests and full reporting of outcomes
☐	☒	Estimates of effect sizes (e.g. Cohen's *d*, Pearson's *r*), indicating how they were calculated

Our web collection on statistics for biologists contains articles on many of the points above.

Software and code

Policy information about availability of computer code

Data collection	We abstracted clinical data from the electronic medical record into standardized case report forms housed in a REDCap database.
Data analysis	We used Cell-Ranger mkfastq (10x Genomics) to demultiplex and produce raw fastq files for downstream analyses. We used Cell-Ranger multi to align per-sample reads of gene expression to the GRCh38 human reference genome, as well as feature barcoded TotalSeq antibodies to their reference sequences. We combined per-sample-count matrices into a filtered aggregated matrix using Cell-Ranger aggr. We excluded cells with < 500 genes, > 4,500 features, >12.5% reads mapping to the mitochondrial genome, > 15% genes mapping to ribosomal genes, and >5% genes aligning to heme-associated genes.<br><br>We used query-reference mapping in Seurat to annotate cells based on their similarity to expression in the provided peripheral blood monocyte dataset. We filtered our Seurat object to only include monocytes, macrophages, and classic dendritic cells as defined by this reference. To account for batch effects, we normalized each sample (method = Center Log Fold Ratio) and filtered based on the above quality control metrics independently before integrating samples via Seurat's FindIntegrationAnchors function (number of variable anchors = 2000).<br><br>We performed PCA analysis in Seurat using both surface antibodies and gene expression data (30 PCs from gene expression and 15 PCs from surface antibodies), followed by dimensional reduction (UMAP, dims: 1:30). We calculated nearest neighbors and performed Louvain clustering in Seurat (resolution = 0.3). We identified both top gene and antibody markers for each of our clusters using FindMarkers (Wilcox-T test, log2fc > 0.25, p < 0.05 for gene expression, p < 0.05 and log2fc > 0.1 for antibodies). In a sensitivity analysis, we performed noise reduction of the antibody markers by utilizing three different mouse isotype negative controls to account for background technical noise generated from factors such as ambient, unbound antibody encapsulated in the droplets. We projected the cell-surface protein "antigen specificity score," which is the likelihood of an antigen binding to a specific receptor compared to the negative control ((1–beta.cdf (0.925, Antigen UMI + 1, Control UMI + 3)) * 100), onto the alveolar myeloid cluster UMAP.

For our integrated blood-lung analysis, we used integration anchors in Seurat to perform query-reference mapping between our BAL dataset and paired PBMC samples. This allowed PBMC samples to be visualized on the previously produced UMAP projection of BAL cells. We annotated our PBMC samples based on cluster identities derived from our BAL dataset (Multimodal Reference Mapping), as well as calculated additional PCs (n = 30) and UMAP (dims: 1:30) to describe variation in the combined PBMC and BAL dataset. We used ggplot2 to visualize our analyses. We used T-tests or Mann-Whitney tests based on whether the data had a parametric or non-parametric distribution for univariate comparisons of expression levels or percent populations. Sample size for power analysis was not predetermined for our study.

We derived average gene expression values for B1 and B2 alveolar myeloid cells via the AverageExpression Function in Seurat, creating pseudo-bulked samples for further differential expression analysis. We then identified differentially expressed genes between B1 and B2 timepoints (log2fc > 0.25, p-value < 0.05). In order to compare our data with previously published data from healthy participants, we batch corrected and integrated both datasets via shared variable genes using FindIntegrationAnchors (n anchors = 2000). We averaged the scaled expression of each gene across our samples and our clusters using the pseudo-bulk approach described above.

We performed RNA velocity and trajectory analyses on the BAL samples by extracting the metadata from our filtered Seurat object and used it to construct an anndata object for processing in python using Scampy, NumPy, and pandas. Loom files containing spliced and unspliced reads on a per sample basis were constructed using velocyto, and integrated with our pre-existing metadata with the merge utility accompanying the package scVelo. We used scVelo to calculate velocity scores for each cell in our dataset, and visualized our previously constructed projections with the included velocity vectors. We generated a PAGA velocity graph using the RNA velocity data. All statistical analyses were performed in R version 4.2.1 and in Python2.

For manuscripts utilizing custom algorithms or software that are central to the research but not yet described in published literature, software must be made available to editors and reviewers. We strongly encourage code deposition in a community repository (e.g. GitHub). See the Nature Portfolio guidelines for submitting code & software for further information.

Data

Policy information about availability of data

All manuscripts must include a data availability statement. This statement should provide the following information, where applicable:

- Accession codes, unique identifiers, or web links for publicly available datasets
- A description of any restrictions on data availability
- For clinical datasets or third party data, please ensure that the statement adheres to our policy

We have deposited our CITE-seq data into the GEO database (accession number: GSE234918). We have deposited our code in GitHub (https://github.com/BenaroyaResearch/Alveolar_Macrophage_Subsets_ARDS).

Research involving human participants, their data, or biological material

Policy information about studies with human participants or human data. See also policy information about sex, gender (identity/presentation), and sexual orientation and race, ethnicity and racism.

Reporting on sex and gender	Reporting on sex and gender is described in Table 1. We did not stratify our analyses by sex or gender due to the sample size.
Reporting on race, ethnicity, or other socially relevant groupings	Reporting on race and ethnicity is described in Table 1. We did not stratify our analyses by race or ethnicity due to the sample size.
Population characteristics	Population characteristics are reported in Table 1 and Table 2.
Recruitment	Research coordinators at Harborview Medical Center screened the electronic medical record for potential participants who met our study's inclusion and exclusion criteria (Table S1). The legal next of kin of potential participants who met study inclusion/exclusion criteria were approached by research coordinators to obtain informed consent to participant in the study. If legal next of kin consented for the study, participants were enrolled.
Ethics oversight	The University of Washington Human Subjects Division provided ethics oversight for this study.

Note that full information on the approval of the study protocol must also be provided in the manuscript.

Field-specific reporting

Please select the one below that is the best fit for your research. If you are not sure, read the appropriate sections before making your selection.

[x] Life sciences [ ] Behavioural & social sciences [ ] Ecological, evolutionary & environmental sciences

For a reference copy of the document with all sections, see nature.com/documents/nr-reporting-summary-flat.pdf

Life sciences study design

All studies must disclose on these points even when the disclosure is negative.

Sample size	Sample size for power analysis was not predetermined for our study.

Data exclusions	None
Replication	We were able to validate CD14, CD71, and CD163 in our HMC Clinical Cohort using flow cytometry because that cell-surface antibody panel contained these antibody markers. We were not able to validate the other cell-surface proteins that our CITE-seq data identified because the flow cytometry data was generated prior to the CITE-seq experiments.
Randomization	This is a cohort study testing whether alveolar leukocyte subsets are associated with clinical outcomes and other biologic measures. Therefore, randomization into intervention groups is not applicable.
Blinding	Research scientists who performed the CITE-seq and flow cytometry experiments were blinded to clinical data. The computation core responsible for the sequencing and alignment pipeline did not have access to any clinical information. The computational biologist who performed all computation analyses, including clustering and subset identification, did not have access to any clinical information. The clinicians who generated the clinical datasets did not have access to any of the raw molecular data.

Reporting for specific materials, systems and methods

We require information from authors about some types of materials, experimental systems and methods used in many studies. Here, indicate whether each material, system or method listed is relevant to your study. If you are not sure if a list item applies to your research, read the appropriate section before selecting a response.

Materials & experimental systems

n/a	Involved in the study	
☐	☒	Antibodies
☒	☐	Eukaryotic cell lines
☒	☐	Palaeontology and archaeology
☒	☐	Animals and other organisms
☐	☒	Clinical data
☒	☐	Dual use research of concern
☒	☐	Plants

Methods

n/a	Involved in the study	
☒	☐	ChIP-seq
☐	☒	Flow cytometry
☒	☐	MRI-based neuroimaging

Antibodies

Antibodies used

Please refer to Table S3 for full details:

CD86 anti-human CD86 IT2.2 GTCTTTGTCAGTGCA
CD274 anti-human CD274 (B7-H1, PD-L1) 29E.2A3 GTTGTCCGACAATAC
TNFRSF14 anti-human CD270 (HVEM, TR2) 122 TGATAGAAACAGACC
PVR anti-human CD155 (PVR) SKII.4 ATCACATCGTTGCCA
NECTIN2 anti-human CD112 (Nectin-2) TX31 AACCTTCCGTCTAAG
CD47 anti-human CD47 CC2C6 GCATTCTGTCACCTA
CD48 anti-human CD48 BJ40 CTACGACGTAGAAGA
CD40 anti-human CD40 5C3 CTCAGATGGAGTATG
CD40LG anti-human CD154 24-31 GCTAGATAGATGCAA
CD52 anti-human CD52 HI186 CTTTGTACGAGCAAA
CD3D anti-human CD3 UCHT1 CTCATTGTAACTCCT
CD8A anti-human CD8 SK1 GCGCAACTTGATGAT
NCAM1 anti-human CD56 5.1H11 TCCTTTCCTGATAGG
CD19 anti-human CD19 HIB19 CTGGGCAATTACTCG
CD33 anti-human CD33 P67.6 TAACTCAGGGCCTAT
ITGAX anti-human CD11c S-HCL-3 TACGCCTATAACTTG
HLA-A anti-human HLA-A,B,C W6/32 TATGCGAGGCTTATC
PTPRC anti-human CD45RA HI100 TCAATCCTTCCGCTT
IL3RA anti-human CD123 6H6 CTTCACTCTGTCAGG
CD7 anti-human CD7 CD7-6B7 TGGATTCCCGGACTT
ENG anti-human CD105 43A3 ATCGTCGAGAGCTAG
ITGA6 anti-human/mouse CD49f GoH3 TTCCGAGGATGATCT
CCR4 anti-human CD194 (CCR4) L291H4 AGCTTACCTGCACGA
CD4 anti-human CD4 RPA-T4 TGTTCCCGCTCAACT
CD44 anti-mouse/human CD44 IM7 TGGCTTCAGGTCCTA
CD14 anti-human CD14 M5E2 TCTCAGACCTCCGTA
FCGR3A anti-human CD16 3G8 AAGTTCACTCTTTGC
IL2RA anti-human CD25 BC96 TTTGTCCTGTACGCC
PTPRC anti-human CD45RO UCHL1 CTCCGAATCATGTTG
PDCD1 anti-human CD279 (PD-1) EH12.2H7 ACAGCGCCGTATTTA

TIGIT anti-human TIGIT (VSTM3) A15153G TTGCTTACCGCCAGA
isotype Mouse IgG1, κ isotype Ctrl MOPC-21 GCCGGACGACATTAA
isotype Mouse IgG2a, κ isotype Ctrl MOPC-173 CTCCTACCTAAACTG
isotype Mouse IgG2b, κ isotype Ctrl MPC-11 ATATGTATCACGCGA
isotype Rat IgG2b, κ Isotype Ctrl RTK4530 GATTCTTGACGACCT
MS4A1 anti-human CD20 2H7 TTCTGGGTCCCTAGA
NCR1 anti-human CD335 (NKp46) 9E2 ACAATTTGAACAGCG
PECAM1 anti-human CD31 WM59 ACCTTTATGCCACGG
MCAM anti-human CD146 P1H12 CCTTGGATAACATCA
IGHM anti-human IgM MHM-88 TAGCGAGCCCGTATA
CD5 anti-human CD5 UCHT2 CATTAACGGGATGCC
CXCR3 anti-human CD183 (CXCR3) G025H7 GCGATGGTAGATTAT
CCR5 anti-human CD195 (CCR5) J418F1 CCAAAGTAAGAGCCA
FCGR2A anti-human CD32 FUN-2 GCTTCCGAATTACCG
CCR6 anti-human CD196 (CCR6) G034E3 GATCCCTTTGTCACT
CXCR5 anti-human CD185 (CXCR5) J252D4 AATTCAACCGTCGCC
ITGAE anti-human CD103 (Integrin αE) Ber-ACT8 GACCTCATTGTGAAT
CD69 anti-human CD69 FN50 GTCTCTTGGCTTAAA
SELL anti-human CD62L DREG-56 GTCCCTGCAACTTGA
KLRB1 anti-human CD161 HP-3G10 GTACGCAGTCCTTCT
CTLA4 anti-human CD152 (CTLA-4) BNI3 ATGGTTCACGTAATC
LAG3 anti-human CD223 (LAG-3) 11C3C65 CATTTGTCTGCCGGT
KLRG1 anti-human KLRG1 (MAFA) SA231A2 CTTATTTCCTGCCCT
CD27 anti-human CD27 O323 GCACTCCTGCATGTA
LAMP1 anti-human CD107a (LAMP-1) H4A3 CAGCCCACTGCAATA
FAS anti-human CD95 (Fas) DX2 CCAGCTCATTAGAGC
TNFRSF4 anti-human CD134 (OX40) Ber-ACT35 (ACT35) AACCCACCGTTGTTA
HLA-DRA anti-human HLA-DR L243 AATAGCGAGCAAGTA
CD1C anti-human CD1c L161 GAGCTACTTCACTCG
ITGAM anti-human CD11b ICRF44 GACAAGTGATCTGCA
FCGR1A anti-human CD64 10.1 AAGTATGCCCTACGA
THBD anti-human CD141 (Thrombomodulin) M80 GGATAACCGCGCTTT
CD1D anti-human CD1d 51.1 TCGAGTCGCTTATCA
KLRK1 anti-human CD314 (NKG2D) 1D11 CGTGTTTGTTCCTCA
CR1 anti-human CD35 E11 ACTTCCGTCGATCTT
B3GAT1 anti-human CD57 Recombinant QA17A04 AACTCCCTATGGAGG
BTLA anti-human CD272 (BTLA) MIH26 GTTATTGGACTAAGG
ICOS anti-human/mouse/rat CD278 (ICOS) C398.4A CGCGCACCCATTAAA
CD58 anti-human CD58 (LFA-3) TS2/9 GTTCCTATGGACGAC
ENTPD1 anti-human CD39 A1 TTACCTGGTATCCGT
CX3CR1 anti-human CX3CR1 K0124E1 AGTATCGTCTCTGGG
CD24 anti-human CD24 ML5 AGATTCCTTCGTGTT
CR2 anti-human CD21 Bu32 AACCTAGTAGTTCGG
ITGAL anti-human CD11a TS2/4 TATATCCTTGTGAGC
CD79B anti-human CD79b (Igβ) CB3-1 ATTCTTCAACCGAAG
CD244 anti-human CD244 (2B4) C1.7 TCGCTTGGATGGTAG
SIGLEC1 anti-human CD169 (Sialoadhesin, Siglec-1) 7-239 TACTCAGCGTGTTTG
ITGB7 anti-human/mouse integrin β7 FIB504 TCCTTGGATGTACCG
TNFRSF13C anti-human CD268 (BAFF-R) 11C1 CGAAGTCGATCCGTA
GP1BB anti-human CD42b HIP1 TCCTAGTACCGAAGT
ICAM1 anti-human CD54 HA58 CTGATAGACTTGAGT
SELP anti-human CD62P (P-Selectin) AK4 CCTTCCGTATCCCTT
IFNGR1 anti-human CD119 (IFN-γ R α chain) GIR-208 TGTGTATTCCCTTGT
isotype Rat IgG1, κ isotype Ctrl RTK2071 ATCAGATGCCCTCAT
Isotype Rat IgG2a, κ Isotype Ctrl RTK2758 AAGTCAGGTTCGTTT
isotype Armenian Hamster IgG Isotype Ctrl HTK888 CCTGTCATTAAGACT
IL2RB anti-human CD122 (IL-2Rβ) TU27 TCATTTCCTCCGATT
TNFRSF13B anti-human CD267 (TACI) 1A1 AGTGATGGAGCGAAC
FCER1A anti-human FcεRIα AER-37 (CRA-1) CTCGTTTCCGTATCG
ITGA2B anti-human CD41 HIP8 ACGTTGTGGCCTTGT
TNFRSF9 anti-human CD137 (4-1BB) 4B4-1 CAGTAAGTTCGGGAC
CD163 anti-human CD163 GHI/61 GCTTCTCCTTCCTTA
CD83 anti-human CD83 HB15e CCACTCATTTCCGGT
IL4R anti-human CD124 (IL-4Rα) G077F6 CCGTCCTGATAGATG
ANPEP anti-human CD13 WM15 TTTCAACGCCCTTTC
CD2 anti-human CD2 TS1/8 TACGATTTGTCAGGG
CD226 anti-human CD226 (DNAM-1) 11A8 TCTCAGTGTTTGTGG
ITGB1 anti-human CD29 TS2/16 GTATTCCCTCAGTCA

CLEC4C anti-human CD303 (BDCA-2) 201A GAGATGTCCGAATTT
ITGA2 anti-human CD49b P1E6-C5 GCTTTCTTCAGTATG
CD81 anti-human CD81 (TAPA-1) 5A6 GTATCCTTCCTTGGC
IGHD anti-human IgD IA6-2 CAGTCTCCGTAGAGT
ITGB2 anti-human CD18 TS1/18 TATTGGGACACTTCT
CD28 anti-human CD28 CD28.2 TGAGAACGACCCTAA
CD38 anti-human CD38 HIT2 TGTACCCGCTTGTGA
IL7R anti-human CD127 (IL-7Rα) A019D5 GTGTGTTGTCCTATG
PTPRC anti-human CD45 HI30 TGCAATTACCCGGAT
CD22 anti-human CD22 S-HCL-1 GGGTTGTTGTCTTTG
TFRC anti-human CD71 CY1G4 CCGTGTTCCTCATTA
DPP4 anti-human CD26 BA5b GGTGGCTAGATAATG
CD36 anti-human CD36 5-271 TTCTTTGCCTTGCCA
KIR2DL1 anti-human CD158 (KIR2DL1/S1/S3/S5) HP-MA4 TATCAACCAACGCTT
ITGA1 anti-human CD49a TS2/7 ACTGATGGACTCAGA
ITGA4 anti-human CD49d 9F10 CCATTCAACTTCCGG
NT5E anti-human CD73 (Ecto-5'-nucleotidase) AD2 CAGTTCCTCAGTTCG
OLR1 anti-human LOX-1 15C4 ACCCTTTACCGAATA
KIR2DL3 anti-human CD158b (KIR2DL2/L3, NKAT2) DX27 GACCCGTAGTTTGAT
KIR3DL1 anti-human CD158e1 (KIR3DL1, NKB1) DX9 GGACGCTTTCCTTGA
SLAMF7 anti-human CD319 (CRACC) 162.1 AGTATGCCATGTCTT
CD99 anti-human CD99 3B2/TA8 ACCCGTCCCTAAGAA
CLEC12A anti-human CLEC12A 50C1 CATTAGAGTCTGCCA
SLAMF6 anti-human CD352 (NTB-A) NT-7 AGTTTCCACTCAGGC
KLRD1 anti-human CD94 DX22 CTTTCCGGTCCTACA
IGKC anti-human Ig light chain κ MHK-49 AGCTCAGCCAGTATG
LILRB1 anti-human CD85j (ILT2) GHI/75 CCTTGTGAGGCTATG
FCER2 anti-human CD23 EBVCS-5 TCTGTATAACCGTCT
SIGLEC7 anti-human CD328 (Siglec-7) 6-434 CTTAGCATTTCACTG
ADGRG1 anti-human GPR56 CG4 GCCTAGTTTCCGTTT
HLA-E anti-human HLA-E 3D12 GAGTCGAGAAATCAT
CD82 anti-human CD82 ASL-24 TCCCACTTCCGCTTT
CD101 anti-human CD101 (BB27) BB27 CTACTTCCCTGTCAA
C5AR1 anti-human CD88 (C5aR) S5/1 GCCGCATGAGAAACA
GGT1 anti-human CD224 KF29 CTGATGAGATGTCAG
CD206 anti-human CD206 15-2 TCAGAACGTCTAACT
CD298 anti-human CD298 (PD1) EH12.2H7 ACAGCGCCGTATTTA
CCR2 Anti-human CCR2 K036C2 GAGTTCCCTTACCTG
Hashtag 9 Hashtag 9 LNH-94;2M2 CAGTAGTCACGGTCA
Hashtag 13 Hashtag 13 LNH-94; 2M2 AAATCTCTCAGGCTC

Please refer to Table S11 for full details:

CD45 2D1 Alexa Fluor 700
CD3 UCHT1 Brilliant Violet 570
CD11b ICRF44 APC
CD14 M5E2 APC/Cyanine7
CD15 HI98 Brilliant Ultra Violet 395
CD71 CY1G4 Brilliant Violet 650
CD163 GHI/61 Brilliant Violet 605
CD172a 15-414 FITC
CD192 K036C2 PE/Dazzle 594
CD169 7-239 Brilliant Violet 421
CD206 15-2 Brilliant Violet 785
CD274 29E.2A3 Brilliant Violet 711
CD282 TL2.1 PE
CD326 9C4 PE/Cyanine7

Validation

All antibodies we used for this study are commercially available and validated by the companies we purchase them from (e.g. BioLegend, BD Biosciences). We purchased all antibodies directly from established vendors and stored the products as recommended.

Clinical data

Policy information about clinical studies

All manuscripts should comply with the ICMJE guidelines for publication of clinical research and a completed CONSORT checklist must be included with all submissions.

Clinical trial registration	This cohort study was not registered with ClinicalTrials.gov.
Study protocol	The study protocol was described in detail in the Methods section of the manuscript. The full study protocol can be provided upon request.
Data collection	This information is provided in the Methods section of the manuscript.
Outcomes	This information is provided in the Methods section of the manuscript.

Flow Cytometry

Plots

Confirm that:

- [x] The axis labels state the marker and fluorochrome used (e.g. CD4-FITC).
- [x] The axis scales are clearly visible. Include numbers along axes only for bottom left plot of group (a 'group' is an analysis of identical markers).
- [x] All plots are contour plots with outliers or pseudocolor plots.
- [x] A numerical value for number of cells or percentage (with statistics) is provided.

Methodology

Sample preparation	This information is provided in the Methods section of the manuscript.
Instrument	This information is provided in the Methods section of the manuscript.
Software	This information is provided in the Methods section of the manuscript.
Cell population abundance	This information is provided in the Methods section of the manuscript.
Gating strategy	This information is provided in the Methods section and Figure 5 of the manuscript.

- [x] Tick this box to confirm that a figure exemplifying the gating strategy is provided in the Supplementary Information.
